# Scimitar syndrome: A rare disease with unusual presentation

**DOI:** 10.4103/0970-2113.45202

**Published:** 2009

**Authors:** Manohar Lal Gupta, Rajeev Bagarhatta, Jyotsna Sinha

**Affiliations:** *Department of Chest and Tuberculosis, SMS Medical College, Jaipur, India*; 1 *Department of Cardiology, SMS Medical College, Jaipur, India*

**Keywords:** Hemoptysis, pulmonary arterial hypertension, scimitar syndrome

## Abstract

Scimitar syndrome is a rare congenital disorder. It has a varied presentation. In adult life, it usually presents either as recurrent chest infection and/or exertional dyspnea. Pulmonary artery hypertension and hemoptysis both are uncommon features of this syndrome in adult life.

## INTRODUCTION

Scimitar syndrome consists of a partial anomalous pulmonary venous drainage of right lung, right lung hypoplasia, dextraposition of heart, and anomalous systemic arterial supply from aorta or one of its branches to the right lung. This syndrome has varied presentations, from an asymptomatic state^1^ to severe pulmonary hypertension and/or heart failure.^2^ Those who present early in life usually have associated congenital heart disease also.^3^ Hemoptysis^4^ and pulmonary arterial hypertension (PAH)^5^,^6^ both are uncommon presenting complaints of this rare syndrome beyond infancy.

Considering the rareness of the syndrome and its unusual form of presentation, this case is reported.

## CASE HISTORY

A 14-year-old girl reported with the complaint of hemoptysis of seven days duration. She denied history of any fever, cough, expectoration, chest pain, and/or any extrapulmonary symptoms. On examination, the patient was comfortable and did not have any lymphadenopathy or clubbing. Her pulse was 86 beats/minute regular, blood pressure 100/70 mm Hg, and respiratary rate 16 breaths/minute.

**Figure 1:**
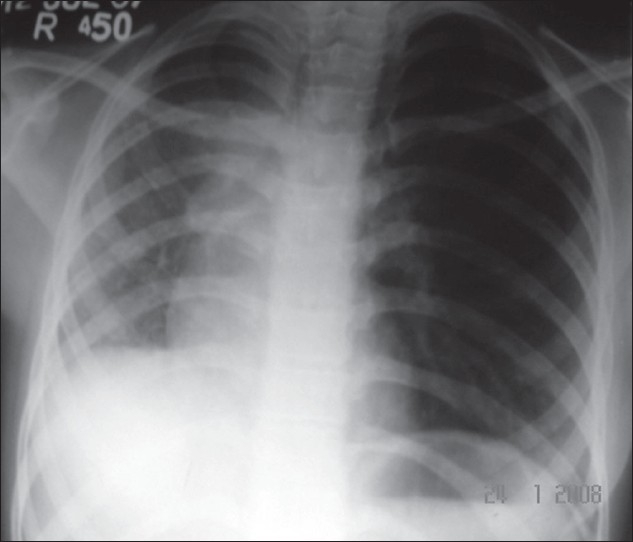
Chest radiograph showing volume loss on the right side, with shift of heart and mediastinum

Respiratory system examination revealed small right hemithorax and reduced intensity of breath sounds on the right side. There were occasional crepitations on the right side of chest.Cardiac examination revealed shift of the apex beat to the right side in fourth intercostal space in right parasternal area without any murmur. Blood counts, bleeding and coagulation profile, and renal and hepatic functions were within normal limits. Skiagram chest PA [Figure 1] showed small right hemithorax with shift of trachea and heart toward right side. Right lung field was more opaque than left due to increased background reticulations.

Electrocardiography was normal except R/S >1 in V_1_. Transesophageal echocardiography in bicaval view showed no evidence of any atrial septal defect. Main pulmonary artery and left pulmonary artery was well seen but right pulmonary artery could not be visualized.

**Figure 2:**
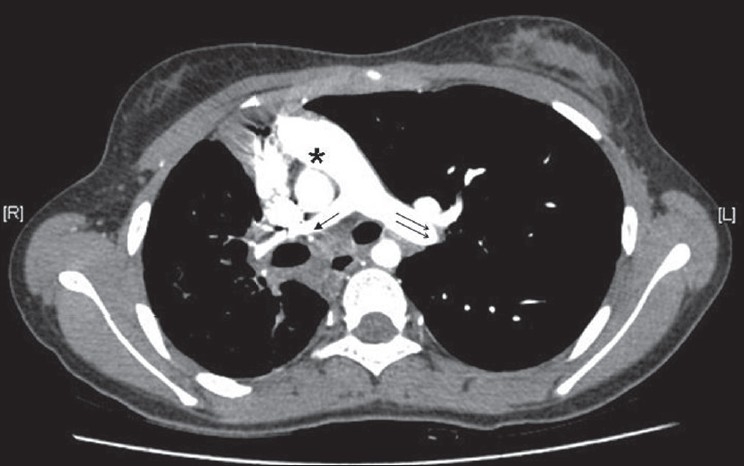
CT thorax showing dilated main pulmonary (asterisk) with normal looking left pulmonary artery (double arrow), and hypoplastic right pulmonary artery (single arrow)

**Figure 3:**
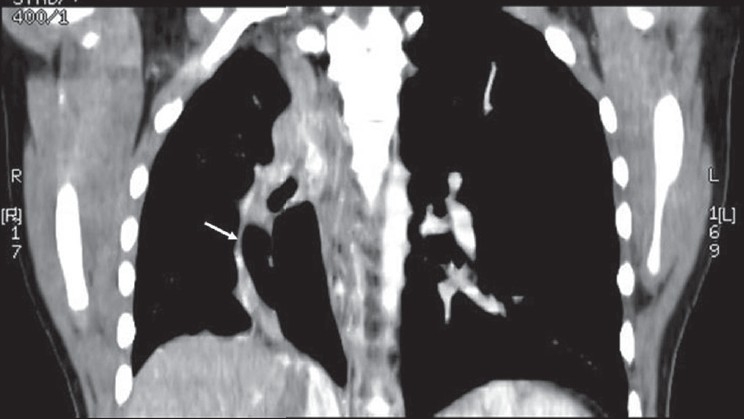
CT thorax showing a curved linear (scimitar shape) opacity running from the middle of the lung toward diaphragm (shown as arrow)

**Figure 4:**
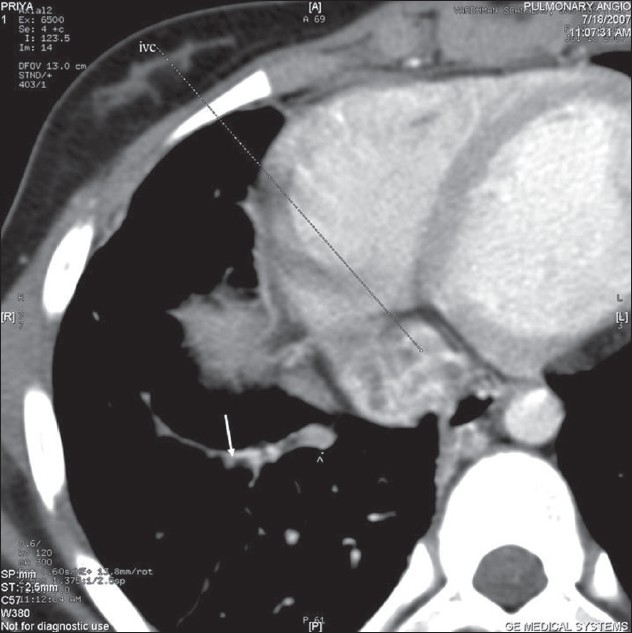
CT thorax showing lower part of the aberrant venous drainage to inferior vena cava (shown as arrow)

**Figure 5:**
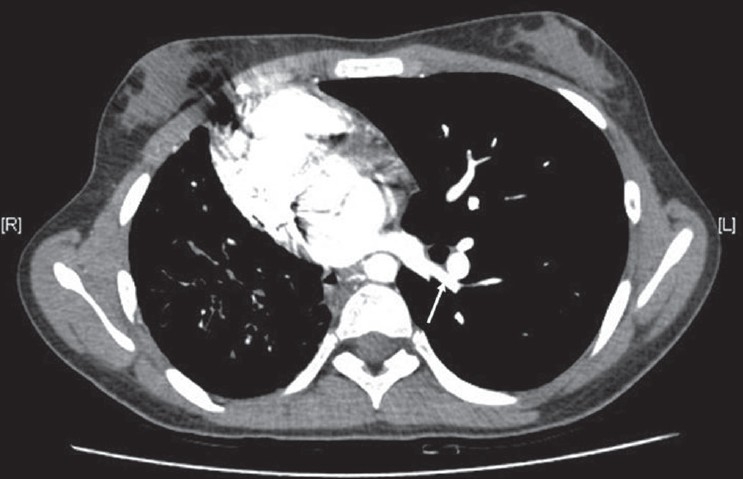
Left pulmonary vein ending into left atrium is well visualized (straight arrow); corresponding pulmonary vein on the right side is absent

**Figure 6:**
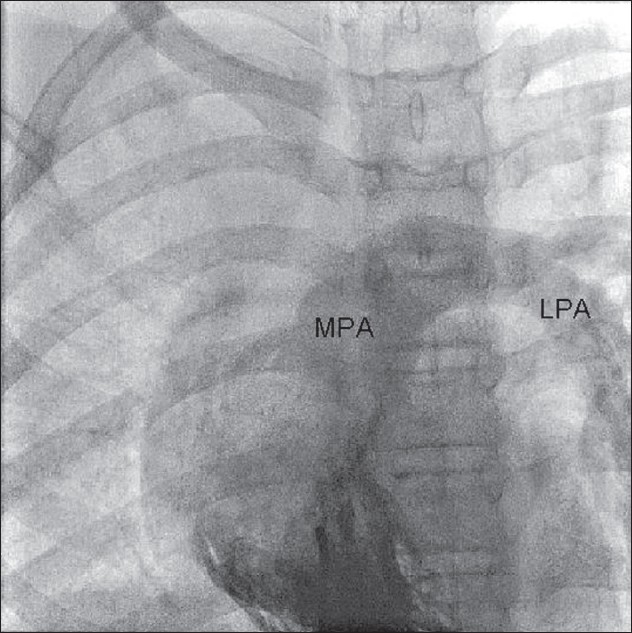
Main pulmonary artery (MPA) and left pulmonary artery (LPA) are filled normally but right pulmonary artery did not fill on right ventriculogram

**Figure 7:**
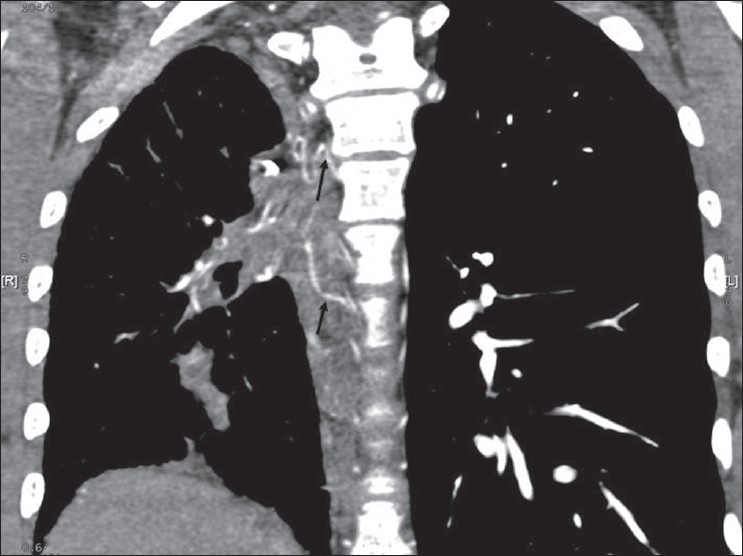
Aortogram showing multiple small curved vessels arising from the thoracic aorta (arrow) and passing toward the hilum

CT angiogram [Figure 2] showed dilated main pulmonary and left pulmonary artery with hypoplastic right pulmonary artery. Coronal reconstruction of the CT study [Figure 3] showed typical scimitar-shaped structure running from the middle of the right lung toward diaphragm. Axial section [Figure 4] at the level of base of the lung showed lower part of scimitar vein running toward the inferior vena cava. Axial cut [Figure 5] at the level of left atrium showed absence of any pulmonary vein ending into the left atrium on the right side. Right ventriculogram [Figure 6] revealed normal filling of main and left pulmonary artery. Right pulmonary artery filling was absent. Aortography [Figure 7] clearly delineated multiple systemic supplies arising from the descending aorta and running toward the right hilum. Cardiac catheterization study demonstrated elevated right ventricular (56/4 mm Hg) and pulmonary artery (56/26 mm Hg) pressures. Aortic and left ventricular pressures were within normal limits. Taking into account the tomography and aortography findings, the diagnosis of scimitar syndrome was made and symptomatic treatment was initiated in the form of cough sedatives and oral ethymsylate. Hemoptysis subsided in next three days. Surgical options were explained to her kindred, to which they refused.

## DISCUSSION

Important differential diagnosis of small lung and small hilar shadow on conventional chest radiograph includes congenital hypoplastic lung, congenital absence of pulmonary artery, atelectasis of a part of the lung, and Macleod's syndrome. The last two conditions are associated with increased lucency of lung fields of the affected side. Systemic collateral circulation in rest of the conditions including scimitar syndrome is associated with reticular opacities in the lung fields. Congenital hypoplastic lung and absent/hypo plastic pulmonary artery disease can be excluded from scimitar syndrome by normal venous drainage to the left atrium.^7^,^8^

Clinical symptomatology of the scimitar syndrome is governed by the age at which the patient presents. Infants having scimitar syndrome present with cyanosis, poor growth, PAH, and often complex cardiac defects, many of them need surgical intervention having a high mortality.^9^,^10^

Disease in older children and adults commonly presents with recurrent pulmonary infections and/or exertional dyspnea. This group of the patients usually has a benign course.^6^

Diagnosis of this syndrome is straightforward in presence of characteristic radiological sign (scimitar sign) on conventional chest radiography. However, when scimitar vein is masked by cardiac shadow, diagnosis can be documented by one or more traditional modalities, for example, angiography,^11^ CT scan,^12^ and echocardiography.^2^ Currently available MR technology also provides excellent visualization of vascular anatomy of this complex congenital defect noninvasively.^13^

Hemoptysis as a presenting symptom is exceptionally rare in patients with scimitar syndrome.^4^,^14^ Among the five large series of patients with this syndrome comprising of total 39 subjects aged one year and above, only four had hemoptysis during their course of illness.^5^,^6^,^11^,^15^,^16^ Interestingly, literature records the youngest patient presenting with hemoptysis to be of the age of seven years.^6^ Possible mechanisms of hemoptysis in scimitar syndrome include rupture of hypertrophied systemic pulmonary anastomosis^17^ or bleeding from some of the bronchiectatic segment in the hypoplastic lung. As HRCT thorax did not show any evidence of bronchiectasis in either lung, former mechanism for hemoptysis seems more likely in the present case.

PAH is a problem of infancy and its presence in a patient aged 14 years is also exceedingly uncommon. Only three out of a total 36 patients aged one year and above from different case reports of scimitar syndrome had PAH.^5^,^6^,^11^,^15^,^16^ Honey^15^ in his case report has reviewed nine cases of PAH having pulmonary arterial pressure exceeding 50 mm Hg available in literature. He observed that pulmonary arterial pressure greater than 50 mm Hg is exceedingly rare in those who present beyond the infancy. Further, he also noted that PAH in this population may also be associated with cardiac abnormalities.

Present case had a greater magnitude of PAH (56 mm Hg) in absence of any concomitant congenital cardiac defect. This could be explained possibly by larger degree of anomalous pulmonary venous drainage, greater extent of hypoplasia of right lung and pulmonary artery, and increased systemic blood flow to the hypoplastic lung.^6^ Lesser magnitude of these abnormalities delays the development of PAH.
